# robustocs: robust optimal contribution selection

**DOI:** 10.1093/bioinformatics/btag569

**Published:** 2026-07-30

**Authors:** Josh Fogg, Jaime Ortiz-Cuadros, Ivan Pocrnic, J A Julian Hall, Gregor Gorjanc

**Affiliations:** The Maxwell Institute, School of Mathematics, The University of Edinburgh, Edinburgh EH9 3FD, United Kingdom; The Roslin Institute and Royal (Dick) School of Veterinary Science, The University of Edinburgh, Midlothian EH25 9RG, United Kingdom; The Roslin Institute and Royal (Dick) School of Veterinary Science, The University of Edinburgh, Midlothian EH25 9RG, United Kingdom; The Maxwell Institute, School of Mathematics, The University of Edinburgh, Edinburgh EH9 3FD, United Kingdom; The Roslin Institute and Royal (Dick) School of Veterinary Science, The University of Edinburgh, Midlothian EH25 9RG, United Kingdom

## Abstract

**Summary:**

Optimal contribution selection (OCS) is a selective breeding method that manages the conversion of genetic variation into genetic gain to facilitate short-term competitiveness and long-term sustainability of breeding programmes. Traditional approaches to OCS and truncation selection (TS) rely on estimates of breeding values and do not explicitly account for uncertainty in these estimates. Here, we use concepts from robust optimization to formulate a robust optimal contribution selection problem (ROCS) and develop two solutions based on conic optimization and sequential quadratic programming. We implemented these methods in the robustocs Python package, which leverages the Gurobi and HiGHS solvers. Our results show favorable performance when solving the ROCS problem using sequential quadratic programming with the HiGHS solver. We show that classical TS and OCS arise as special cases of the robust selection formulations (RTS and ROCS). We demonstrate the package with a small example, comparing outcomes of TS, RTS, OCS, and ROCS. Compared to TS and OCS, RTS and ROCS find contributions that reduce the uncertainty of genetic gain and group coancestry at the expense of reduced genetic gain.

**Availability and implementation:**

robustocs is implemented in Python and released, with documentation, on GitHub under the MIT license at https://github.com/Foggalong/RobustOCS.

## 1 Introduction

In selective breeding programmes it is important to manage the conversion of genetic variation into genetic gain with care. The efficiency of this conversion depends on the optimal use of genetically superior individuals to maximize short- and long-term response to selection (Woolliams *et al.* 2015).

Given a cohort of *n* selection candidates, we approach this problem by first obtaining a breeding value $\mu_{i}$ for each candidate, stored in an *n* -dimensional vector μ, which describes the genetic value of individuals as parents of the next generation. In addition, we obtain a genetic relationship (coancestry) matrix Σ, with coefficient $\sigma_{ij}$ between candidates *i* and *j*, which describes genetic similarity between candidates (Henderson 1976). In optimal contribution selection (OCS) (Meuwissen 1997) we use μ and Σ to find a vector w of the selection candidates’ contributions to the next generation, with $w_{i}$ denoting the contribution of the $i^{\text{th}}$ candidate. Since w is a vector of proportions its entries must sum to one, *i.e.* $\sum_{i}w_{i}=1$. If in addition our cohort is split into male candidates (sires, S) and female candidates (dams, D), with each required to make half the contribution, then the sum-to-one constraint is superseded by two constraints $\sum_{i\in S}^{\;}w_{i}=\frac{1}{2}$ and $\sum_{i\in D}^{\;}w_{i}=\frac{1}{2}$. We may also set upper bounds u and lower bounds l on contributions. Using only μ yields truncation selection (TS), with non-zero contributions for the highest-ranked candidates.

In OCS we maximize response to selection (by assigning higher contributions to candidates with higher breeding values) while minimizing risks due to inbreeding (by assigning lower contributions to closely related candidates) (Meuwissen 1997). This can be formulated as a multi-objective optimization problem:


(1) $$\begin{array}{c} \underset{l\leq w\leq u}{\;\text{min}}\left(\frac{1}{2}w^{T}\Sigma w\right),\;\underset{l\leq w\leq u}{\text{max}}\left(w^{T}\mu \right)\;\text{subject}\;\text{to}\\ \sum_{i\in S}w_{i}=\frac{1}{2}\;\text{and}\;\sum_{i\in D}w_{i}=\frac{1}{2}. \end{array}$$

Since this is a multi-objective problem, we typically reframe it as a single objective maximization problem:


(2) $$\underset{l\leq w\leq u}{\text{max}}\left(w^{T}\mu -\frac{\lambda }{2}w^{T}\Sigma w\right)\;s.t.\;\sum_{i\in S}w_{i}=\frac{1}{2},\;\sum_{i\in D}w_{i}=\frac{1}{2},$$

with a parameter λ≥0 that balances emphasis between the response to selection and risks from inbreeding.

We can solve this using quadratic programming (QP) for varying values of $\lambda^{\left(k\right)}$ to find a set of Pareto optimal solutions $w^{\left(k\right)}$. These are the points where, for the risk corresponding to $w^{\left(k\right)}$, there is no higher genetic merit, and for the genetic merit corresponding to $w^{\left(k\right)}$ there is no lower risk. Higher $\lambda^{\left(k\right)}$ values will correspond to a lower tolerance for risks from inbreeding.

It is helpful to rearrange the problem into “standard form”:


$$\underset{l\leq w\leq u}{\min}\left(w^{T}\Sigma w+q^{T}w\right)\;\text{subject}\;\text{to}\;\mathrm{Gw}\leq h,\mathrm{Mw}=m,$$

with constraints governed by matrices G and M and associated vectors h and m. In doing so, note that the two vector constraints $\sum_{i\in S}^{\;}w_{i}=\frac{1}{2}$ and $\sum_{i\in D}^{\;}w_{i}=\frac{1}{2}$ can be represented as a single matrix constraint Mw=m, where M is a matrix with all entries either 0 or 1, and m is a vector where all entries are $\frac{1}{2}$. The OCS problem does not involve the Gw≤h constraint, though it would be straightforward to adapt the theory to include this. Hence, OCS in standard form is then:


(3) $$\underset{l\leq w\leq u}{\text{max}}\left(w^{T}\mu -\frac{\lambda }{2}w^{T}\Sigma w\right)\;\text{subject}\;\text{to}\;\mathrm{Mw}=m.$$

Similarly, the theory presented may easily be adapted for use with single-sex cohorts or to add general linear constraints, for example of the form Aw=b, where A is some matrix and b some vector.

## 2 Incorporating uncertainty

Breeding values for a given trait are estimated using information on phenotypes and genetic relationships among individuals in a population (Mrode and Pocrnic 2023). This estimation involves calculating the conditional expectation $\bar{\mu }=E(\mu |y)$, corresponding to the estimated breeding values (EBVs), and the associated prediction error covariance (PEC) matrix, given by the conditional covariance Ω=Var(μ∣y), where y is a vector of observed phenotypes. Expectations and covariances are taken with respect to the assumed estimation model. In a multi-trait setting, μ would be the estimated index values and Ω the corresponding prediction error covariance matrix. These conditional quantities can be obtained using closed-form, iterative, or sampling approaches (e.g. García-Cortés *et al.* 1995, Fouilloux and Laloë 2001, Mrode and Pocrnic 2023). The conditional variance decreases as more information becomes available over time. At the time of selection, the amount of information is usually limited, and hence there is uncertainty associated with the estimates. In addition, conditioning on observed phenotypes induces non-zero covariances. Thus any solution to (1) or (2) is contingent on the implicit assumption that the values of μ and Σ are known exactly; in particular, we implicitly assume that $\left(\bar{\mu }\right)$ are equal to true breeding values (μ). While unbiased EBVs will provide an unbiased estimate of $w^{T}\mu$, the optimization of w does not take into account the uncertainty in EBVs. Pruzzo *et al.* (2003) have studied a similar problem and derived a risk-adjusted economic return under TS, where they adjusted the EBV of selection candidates for the corresponding square root of the diagonal of Ω. Here, we generalize this problem by taking into account the uncertainty and correlation of EBV in OCS and TS.

We use robust optimization to address this problem. To this end, we reformulate (3) as a bilevel optimization problem:


$$\underset{l\leq w\leq u}{\text{max}}\left(\underset{\mu \in U_{\mu }}{\text{min}}\left(w^{T}\mu \right)-\frac{\lambda }{2}w^{T}\Sigma w\right)\;\text{subject}\;\text{to}\;Mw=m,$$

where $U_{\mu }$ is some uncertainty set of values for μ that we deem allowable. In bilevel optimization this is called a follower-leader setup. We solve the inner problem first, with its solution impacting the outer problem.

While bilevel optimization is a wide area of research, in our setup we can solve the inner problem explicitly based on our choice of $U_{\mu }$ (Yin *et al.* 2021). For practical reasons relating to continuity and differentiability, we define a ‘quadratic’ uncertainty set:


$$U_{\mu }\left\{\mu :\;{\left(\mu -\bar{\mu }\right)}^{T}\Omega^{-1}(\mu -\bar{\mu })\leq \kappa^{2}\right\},$$

which bounds the uncertainty in a ball about $\bar{\mu }$, with parameter κ controlling the tolerance for uncertainty.

Using the quadratic uncertainty set means that the inner problem ${\text{min}}_{\mu \in U_{\mu }}(w^{T}\mu )$ in standard form becomes:


(4) $$\underset{\mu }{\min}\left(w^{T}\mu \right)\;\text{subject}\;\text{to}\;\kappa^{2}-{\left(\mu -\bar{\mu }\right)}^{T}\Omega^{-1}(\mu -\bar{\mu })\geq 0.$$

This problem is convex because Ω is a positive definite matrix and $w^{T}\mu$ is a linear function. With these conditions met, the first-order necessary conditions (Karush–Kuhn–Tucker) are necessary and sufficient for optimality. If we define a Lagrange multiplier ρ∈R, the conditions for this problem are:


(5) $$\begin{array}{c} \nabla_{\mu }L(\mu ,\rho )=0\;\Rightarrow \\ \nabla_{\mu }\left(w^{T}\mu -\rho (\kappa^{2}-{\left(\mu -\bar{\mu }\right)}^{T}\Omega^{-1}(\mu -\bar{\mu }))\right)=0, \end{array}$$

(6) $$\begin{array}{cc} c(\mu )\geq 0 & \Rightarrow \;\kappa^{2}-{\left(\mu -\bar{\mu }\right)}^{T}\Omega^{-1}(\mu -\bar{\mu })\geq 0,\\ \rho \geq 0 & \Rightarrow \;\rho \geq 0,\\ \rho \cdot c(\mu )=0 & \Rightarrow \;\rho \left(\kappa^{2}-{\left(\mu -\bar{\mu }\right)}^{T}\Omega^{-1}(\mu -\bar{\mu })\right)=0. \end{array}$$

From (5) we have that:


(7) $$w+\rho 2\Omega^{-1}(\mu -\bar{\mu })=0\;\Rightarrow \;\mu -\bar{\mu }=-\frac{1}{2\rho }\Omega w,$$

which when substituted into (6) gives:


$$\begin{array}{cc} & \;\;\rho \left(\kappa^{2}-{\left(-\frac{1}{2\rho }\Omega w\right)}^{T}\Omega^{-1}(-\frac{1}{2\rho }\Omega w)\right)=0\\ & \Rightarrow \;\frac{1}{2\rho }=\frac{\kappa }{\sqrt{w^{T}\Omega w}}. \end{array}$$

Substituting this back into (7), we find that:


$$\mu -\bar{\mu }=-\frac{\kappa }{\sqrt{w^{T}\Omega w}}\Omega w\;\Rightarrow \;\mu =\bar{\mu }-\frac{\kappa }{\sqrt{w^{T}\Omega w}}\Omega w$$

is the solution for the inner problem. After substituting this back into the outer problem and rationalizing, we obtain:


(8) $$\underset{l\leq w\leq u}{\text{max}}\left(w^{T}\bar{\mu }-\kappa \sqrt{w^{T}\Omega w}-\frac{\lambda }{2}w^{T}\Sigma w\right)\;s.t.\;\mathrm{Mw}=m,$$

where κ∈R is the robust optimization parameter controlling the tolerance for uncertainty. Observe that if the κ=0, the robust problem reduces to the standard non-robust problem. Since our objective has gained a square root term, (8) is no longer a quadratic problem.

## 3 Direct conic optimizations

Working with this new form (8) is not ideal, and short of using a general non-linear solver, it is not a problem most off-the-shelf software can handle. However, we can make it tractable by adapting it into the form of a conic optimization problem. If we define a real auxiliary variable z≥0 such that $z\geq \sqrt{w^{T}\Omega w}$, then the problem becomes:


$$\underset{\overset{l\leq w\leq u,}{z\geq 0}}{\text{max}}\left(w^{T}\bar{\mu }-\kappa z-\frac{\lambda }{2}w^{T}\Sigma w\right)\;s.t.\;z\geq \sqrt{w^{T}\Omega w},\;\mathrm{Mw}=m.\;$$

Since z>0 , κ≥0, and we are maximizing an objective containing “−κz,” this term of the objective will be biggest when *z* is smallest. This happens precisely when it attains its lower bound from the constraint $z\geq \sqrt{w^{T}\Omega w}$, hence *z* will push downwards and our relaxation is justified.

However, the presence of the square root still proves problematic for most optimization software, so we further make use of both *z* and $\sqrt{w^{T}\Omega w}$ being positive to note that $z\geq \sqrt{w^{T}\Omega w}$ can be squared on both sides:


(9) $$\underset{\overset{l\leq w\leq u,}{z\geq 0}}{\text{max}}\left(w^{T}\bar{\mu }-\kappa z-\frac{\lambda }{2}w^{T}\Sigma w\right)\;s.t.\;z^{2}\geq w^{T}\Omega w,\;\mathrm{Mw}=m.\;$$

Here $z^{2}\geq w^{T}\Omega w$ represents a cone constraint, hence we are solving a second order conic optimization problem. This opens up the possibility of using standard optimization software, such as the Gurobi’s widely used modeling language (Gurobi Optimization, LLC 2024).

## 4 Sequential quadratic programming

For our other approach, from (9) we note $f(w)=\sqrt{w^{T}\Omega w}$ is a cone with a derivative discontinuity at the origin. For w≠0 (which we can guarantee since $w_{i}\geq 0$ and $\sum_{i}w_{i}=1$) we have:


$$\nabla f(w)=\frac{1}{\sqrt{w^{T}\Omega w}}\Omega w.$$

This means we can approximate the cone using a series of tangent planes (with the $k^{\text{th}}$ denoted $P_{k}$) of the form:


$$z\geq \frac{1}{\sqrt{w_{\left(k\right)}^{T}\Omega w_{\left(k\right)}}}{\left(\Omega w_{\left(k\right)}\right)}^{T}w,$$

for a set of *K* points $w_{\left(0\right)},w_{\left(1\right)},\ldots ,w_{\left(K\right)}$. To find $w_{\left(K\right)}$ we use sequential quadratic programming (SQP), which solves the quadratic problem:


(10) $$\begin{array}{c} \begin{array}{c} \underset{\overset{l\leq w\leq u,}{z\geq 0}}{\text{max}}\left(w^{T}\bar{\mu }-\kappa z-\frac{\lambda }{2}w^{T}\Sigma w\right)\;s.t.\;\mathrm{Mw}=m,\\ z\geq \frac{1}{\sqrt{w_{\left(k\right)}^{T}\Omega w_{\left(k\right)}}}{\left(\Omega w_{\left(k\right)}\right)}^{T}w\;\;\text{for}\;\;k=0,\ldots ,(K-1). \end{array} \end{array}$$

This is a regular (convex) QP problem and, as before, we can use Gurobi’s modeling language Gurobi Optimization, LLC (2024). An important advantage of SQP is that it only relies on QP, so a greater number of solvers may be used.

## 5 Implementation in Python

Though freely available to academics, Gurobi Optimization, LLC (2024) is commercial optimization software with license fees, which are prohibitive to many stakeholders. To democratize this work, we provide a solution that leverages open-source software. One such tool is HiGHS (Huangfu and Hall 2018), released under the MIT license. It can solve convex QP problems, and is applicable to robust OCS using SQP (10).

For ease of use and testing, we implemented these methods as the Python package robustocs, available via PyPI. It accesses the Gurobi API using gurobipy and the HiGHS API using highspy, also using numpy for standard linear algebra tools and scipy for handling sparse matrix objects. The robustocs package is released under the MIT license, with development and documentation at github.com/Foggalong/robustocs.

Suppose we have a breeding cohort with genetic relationship matrix Σ and EBV vector $\bar{\mu }$ with associated covariance matrix Ω. If these are saved in files of appropriate formats, solving the robust OCS using robustocs can be done by running the code below in Python. robustocs is also capable of taking inputs via NumPy or SciPy objects and has more granular functions to control the solver, method, and associated parameters (e.g., maximum solve time).

import robustocs as rocs selection, obj, merit, coancestry = rocs.solveROCS**(** 

sigma_filename = **“**cohort—relationships.txt**“**,

mu_filename = **“**breeding—means.txt**“**,

omega_filename = **“**breeding—variances.txt**“**,

sex_filename = **“**cohort—sexes.txt**“**,

method = “robust“, lam = 0.5, kappa =   *1*

**)** 

Using the simulation outputs, we first compared how the methods performed across Gurobi and HiGHS as the size of the cohort increased. We measured execution time on a HP Elitebook with 15.0 GiB of memory and an Intel® Core™ i5-8350U CPU @ 1.70 GHz × 8, running Ubuntu 22.04.4 LTS (64-bit) with packages robustocs 0.2.1, gurobipy 11.0.3, highspy 1.7.2, numpy 1.21, and scipy 1.8.0. The results in Table 1 show favorable execution time for using HiGHS compared to Gurobi for the standard OCS problem (3). For the robust OCS problem solved with conic optimization (9), Gurobi crashed without displaying an error message. Lastly, HiGHS outperformed Gurobi by an order of magnitude when solving the robust OCS problem with SQP (10), and proved to be a scalable approach.

**Table 1:** Time in seconds to solve standard or robust OCS problems with each method implemented with Gurobi or HiGHS against increasing problem size: (3) is standard OCS, (9) is robust OCS using conic optimization, and (10) is robust OCS using SQP.

Size *n*	Gurobi (3)	HiGHS (3)	Gurobi (9)	Gurobi (10)	HiGHS (10)
4	0.003	0.000	0.005	0.017	0.006
50	0.004	0.001	0.010	0.055	0.018
1000	0.676	0.204	2.750	26.400	1.680
10 000	86.300	25.800	DNF	1560.000	106.000

## 6 Demonstration

To demonstrate the methods, we simulated an example dataset using AlphaSimR (Gaynor *et al.* 2021) with 10 cattle-like chromosomes and a complex trait with heritability of 0.3 affected by 10 000 causal loci. The simulation mimicked a breeding programme over 10 generations with 1000 individuals in each. In each generation, we selected the best 25 males (out of 500) as fathers based on their phenotypes and mated them with all 500 females from the previous generation and all 500 females from the current generation. These matings produced 1000 selection candidates for the next generation, in total 10 000 across 10 generations. We collected phenotypes for all the 10 000 individuals, their pedigree, and 20 000 biallelic marker loci. We fitted pedigree-based and genome-based linear mixed models to the simulated data, with the known Σ defined from pedigree or from marker data (Mrode and Pocrnic 2023). We obtained 1000 samples from the posterior distribution p(μ|y) to estimate $\bar{\mu }$ and Ω from each of the models, providing pedigree EBV (PEBV) and genome EBV (GEBV) and associated covariance matrices. While Ω is also estimated, modeling uncertainty in this quantity is beyond the scope of this study and will likely have diminishing returns.

We then explored how the choice of different λ and κ values impact the results. By setting (λ,κ) as follows we obtain the classic truncation selection (TS) with (0, 0), the novel robust truncation selection (RTS) with (0, 1), the classic optimal contribution selection (OCS) with (1, 0), and the novel robust optimal contribution selection (ROCS) with (1, 1). Since κ penalizes the number of standard deviations of the expected genetic gain $\sqrt{w^{T}\Omega w}$, it controls the risk of breeding decisions (Pruzzo *et al.* 2003, Yin *et al.* 2021), similar to the λ parameter. More research is needed to find useful ranges of values for κ. Our preliminary results show that increasing κ asymptotically reduces expected genetic gain and its error while also reducing group coancestry. The results in Table 2 show realized expected genetic mean $\left(w^{T}\mu \right)$, its standard deviation (SD), the standard error $\left(\sqrt{w^{T}\Omega w}\right)$, and group coancestry $\left(w^{T}\Sigma w\right)$ for these four breeding strategies. With TS, the expected genetic mean, SD, and group coancestry for PEBV are respectively 0.34, 0.24, and 0.18, while using GEBV gave a higher mean of 0.52, a lower SD of 0.16, and a lower group coancestry of 0.13, as expected. With RTS, the SD and group coancestry decreased by approximately half for both PEBV and GEBV, although this came at the cost of a reduced genetic mean. With OCS, we observed comparable changes to RTS. Finally, with ROCS, we observed a further decrease in SD and group coancestry as well as genetic mean, but the decrease in genetic mean was less for GEBV than for PEBV. Overall, the robust optimization framework enables breeders to balance the expected genetic mean, its uncertainty, and group coancestry in line with their breeding strategy and risk management.

**Table 2:** Expected genetic mean $\left(w^{T}\mu \right)$, its standard deviation $\left(\sqrt{w^{T}\Omega w}\right)$, and group coancestry $\left(w^{T}\Sigma w\right)$ under the classic truncation selection (TS), the novel robust truncation selection (RTS), the classic optimal contribution selection (OCS), and the novel robust optimal contribution selection (ROCS) using pedigree- or genome-based estimates of breeding values (PEBV or GEBV).

			$w^{T}\mu \pm \sqrt{w^{T}\Omega w}\;\;\;\left(w^{T}\Sigma w\right)$	
Method	λ	κ	PEBV	GEBV
TS	0	0	0.34±0.24 (0.18)	0.52±0.16 (0.13)
RTS	0	1	0.29±0.11 (0.07)	0.50±0.08 (0.04)
OCS	1	0	0.31±0.12 (0.08)	0.50±0.07 (0.04)
ROCS	1	1	0.27±0.08 (0.06)	0.48±0.06 (0.04)

## 7 Conclusion

We have developed two robust optimization methods to account for uncertainty in selective breeding, and implemented these in a well-documented Python package robustocs. The package can use HiGHS or Gurobi APIs to solve the problems, with HiGHS demonstrating the better performance. We demonstrate use of the package and how to obtain TS and OCS solutions, as well as their robust counterparts, and how these impact the selection. Future work will test more diverse breeding programmes, and assess the impact of accounting for uncertainty in EBVs on long-term breeding outcomes.
